# Development and Assessment of a Brazilian Pilot Massive Open Online Course in Planetary Health Education: An Innovative Model for Primary Care Professionals and Community Training

**DOI:** 10.3389/fpubh.2021.663783

**Published:** 2021-12-15

**Authors:** Mayara Floss, Carlos Augusto Vieira Ilgenfritz, Ylana Elias Rodrigues, Anna Cláudia Dilda, Ana Paula Borngräber Corrêa, Diego Azevedo Conte de Melo, Enrique Falceto Barros, Carlos Alberto Faerron Guzmán, Erin Devlin, Paulo Hilário Nascimento Saldiva, Su-Ming Khoo, Marcelo Rodrigues Gonçalves, the Planetary Health MOOC Group

**Affiliations:** ^1^Programa de Pós Graduação em Patologia, Universidade de São Paulo, São Paulo, Brazil; ^2^Grupo Hospitalar Conceição, Porto Alegre, Brazil; ^3^TelessaúdeRS-UFRGS, Universidade Federal Do Rio Grande Do Sul, Porto Alegre, Brazil; ^4^World Organization of Family Doctors (WONCA) Working Party on the Environment, Santa Maria Do Herval, Brazil; ^5^Planetary Health Alliance, Harvard T.H. Chan School of Public Health, Boston, MA, United States; ^6^Program Manager, Oregon State University, Corvallis, OR, United States; ^7^Institute of Advanced Studies, Universidade de São Paulo, São Paulo, Brazil; ^8^School of Political Science and Sociology and Ryan Institute, National University of Ireland Galway, Galway, Ireland

**Keywords:** Latin America, climate change, primary healthcare (PHC), community training, massive open online course (MOOC), planetary health

## Abstract

**Introduction:** Planetary health (PH) has emerged as a leading field for raising awareness, debating, and finding solutions for the health impacts of human-caused disruptions to Earth's natural systems. PH education addresses essential questions of how humanity inhabits Earth, and how humans affect, and are affected by, natural systems. A pilot massive open online course (MOOC) in PH was created in Brazil in 2020. This MOOC capitalized on the global online pivot, to make the course accessible to a broader audience. This study describes the process of course creation and development and assesses the impact evaluation data and student outcomes of the PH MOOC.

**Methods:** The PH MOOC pilot was launched in Brazilian Portuguese, using the TelessaúdeRS-UFRGS platform on 4/27/2020 and concluded on 7/19/2020 with a total load of 80 h. It was composed of 8 content modules, pre and post-test, 10 topics in a forum discussion, and an optional action plan. This study analyzes the course database, profile of participants, answers to questionnaires, forum interaction, and action plans submitted.

**Results:** Two thousand seven hundred seventy-seven participants enrolled in the course, of which 1,237 (44.54%) gave informed consent for this study. Of the 1,237 participants who agreed to participate in the research, 614 (49.8%) completed the course, and 569 (92.67%) were accredited by TelessaúdeRS-UFRGS. The majority of the participants were concerned with climate change, trained in the health area, and worked in primary health care in places that lacked ongoing sustainability programs. Two hundred forty-one action plans were submitted, major topics identified were food and nutrition, infectious diseases, and garbage and recycling.

**Discussion:** The use of the PH lens and open perspective of the course centered the need to communicate planetary health topics to individuals. The local plans reflected the motto of “think global and act local.” Brazil presents a context of an unprecedented social, political, and environmental crisis, with massive deforestation, extensive fires, and biomass burning altering the biomes, on top of an ongoing necropolitical infodemic and COVID-19 pandemic. In the face of these multiple challenges, this MOOC offers a timely resource for health professionals and communities, encouraging them to address planetary challenges as fundamental health determinants.

## Introduction

The field of planetary health has emerged as a way to raise awareness, discuss and find solutions regarding the health impacts of human-caused disruptions of Earth's natural systems. It aims to educate, restore, and care for both humans and the planet. Planetary health education addresses essential questions of how humanity inhabits Earth, and how humans affect and are affected by natural systems. A key challenge for students is to understand current and future health risks for humanity and the natural systems on which it depends. A major challenge is how to scale up learning to reach global south primary care professionals. Additionally, with the consequences of climate change appearing dramatically in our everyday life, distance education for planetary health seems like an important and urgent development.

The distance education model is an open, accessible and viable alternative and resource for training health professionals and the general public due to its flexibility, transformation of pedagogical frameworks, innovation, and diversification of models of teaching and learning (1). Climate change action runs through education (2). In addition, through distance education, it is possible to train a larger number of people in a short period. Open Education Resources have stood out with increased quality assurance, certification, and accreditation enhancing open and distance courses and degrees (3). Massive open online courses (MOOCs) have emerged as an important and perhaps transformative tool in higher education, representing a new model for providing educational opportunities to students not formally enrolled in an educational program (4).

There was no study or evidence on MOOCs that set Planetary Health as the central theme, at the time of the writing of this article. In this context, two family medicine trainees of the Grupo Hospitalar Conceição decided to create a MOOC in Planetary Health to expand access to this important subject with evidence-based knowledge for a broader community. The course was initially presented to TelessaúdeRS-UFRGS and Grupo Hospitalar Conceição, and received support from different stakeholders (WONCA Working Party on the Environment, Confederación Iberoamericana de Medicina Familiar, Sociedade Brasileira de Medicina de Família e Comunidade, Grupo de Trabalho em Saúde Planetária da SBMFC, Instituto de Estudos Avançados da Universidade de São Paulo IEA/USP, Grupo de Saúde Planetária do IEA/USP, Rural Seeds, Planetary Health Alliance, and the Global Climate and Health Alliance). The conception and creation of a Planetary Health MOOC was initiated in August 2019. This Planetary Health MOOC pilot was launched in Brazilian Portuguese on 4/27/2020 and concluded on 7/19/2020. In this paper, we describe the creation process and development of this innovative source of planetary health education and describe significant findings of the course evaluation and student outcomes.

## Designing the Course and Methods

Initial interest to create this MOOC arose from the common interests of the Rural and Environment working groups of the World Organization of Family Doctors (WONCA), inspired by the WONCA course “Air Health Train the Trainer Program,” and from the production of the Brazilian Lancet Countdown policymakers briefings on how climate change affected the health of Brazilians in 2018 (5) and 2019 (6). This aligned with Porto Alegre's international symposiums on Planetary Health since 2017 (7). The project started as a proposal by two medical residents as a final project of the Grupo Hospitalar Conceição Family Medicine residency program in June 2019. This course was created through a significant mobilization of voluntary effort by medical residents, over and above the 5 h out of a 60 h clinical work-week that were officially assigned to the project work of designing the MOOC. The need to discuss Planetary Health in a broader way and to make the topic accessible was key for the medical residents.

In August 2019 the project was presented to TelessaúdeRS-UFRGS, and the proponents agreed to coordinate it voluntarily. The TelessaúdeRS-UFRGS is a program within the Federal University of Rio Grande do Sul (UFRGS), in partnership with the Brazilian Ministry of Health, the State Health Secretariat of Rio Grande do Sul (SES/RS), and other institutions. The TelessaúdeRS-UFRGS programme provides outreach clinical support and distance education opportunities for primary health professionals throughout the country (8). It offers teleconsulting, telediagnosis, tele-education, tele-regulation, and support of health centers and information systems. The program also provides peer-reviewed evidence-based protocols and guidelines to accredit and enhance the care provided within the Unified Health System (SUS) (8).

Experts from Brazil and abroad met to develop and review eight modules (Table 1). They were chosen through mutual connections and networking across the institutions. The team was composed of 33 authors and reviewers, not including the audiovisual and administrative staff from TelessaúdeRS-UFRGS. The decision to create the course in Portuguese was based on the course's Brazilian origin. With 270 million speakers (9), Portuguese is one of the most spoken languages in the world. Thus, this resource will help to address the lack of training materials for other Lusophone countries. The course structure was rooted in the best evidence available on planetary health and was divided into eight modules, in addition to introductory and closing modules. The modules' authors were selected according to their expertise on the themes and were from Brazil and Latin America. Content reviewing was carefully structured and designed, with individual reviews for each module and high-level review of the course. Pedagogically, the expertise of the TelessaúdeRS-UFRGS and a broad literature review helped to develop a course that integrated emotional, creative, and affective dimensions, which are key to transformative education (10).

**Table 1:** Planetary health course modules and aims.

**Modules**	**Aims**
Setting module	- Introduction to the course interface, aims, pace, and a brief about Sustainable Development Goals (SDGs) related to planetary health
Module 1—Planetary health and climate change	- Conceptualize Planetary Health - Understand the intersections of Planetary Health in the practice of health professionals - Understand the health impact of climate change
Module 2—Heat waves and heat stress	- Define the concept of heat waves - Recognize signs and symptoms of people suffering from heat stress - Understand the impact of heat waves on health
Module 3—Air pollution and health	- Know the sources and mechanisms responsible for the harmful effects of air pollution on health - Identify populations at risk - Understand the mechanism of health damage in different systems and populations - Understand the concepts: air pollution, pollutants/polluting sources - Recognize the boundary parameters of air pollutants and how the measurement is made
Module 4—Infectious diseases sensitive to climate change	- Understand the definitions of infectious and contagious diseases, and their relationship to planetary health and climate change - Reflect and imagine intervention and control actions that health professionals can take to deal with infectious and contagious diseases in a planetary health context
Module 5—Management, mitigation and adaptation	- Understand the concept of mitigation and adaptation to climate change - Recognize planetary health as an instrument in the administration of health services - Identify risks in health services that aggravate changes and how to mitigate and adapt to them
Module 6—Mental and relational health	- Explore the intersection of mental and relational health with planetary health - Look from the perspective of the systemic approach to the human relationship with the planet - Critically define concepts such as ecological grief, climate, and environmental refugees, the VUCA world, *Alegremia*, and *Buen vivir*
Module 7—Food and planetary health	- Present the historical, social, and political context that involves sustainable food for people and the planet - Critically define what sustainable food is, as well as other relevant related concepts - Propose appropriate attitudes for the maintenance of healthy eating and planetary health through recommendations at individual, collective, and institutional levels
Module 8—Amazon case study	- A webinar discussion about the Amazon Forest and the intersections with Planetary Health - The students voluntarily could submit a Planetary Health project for colleague peer review
Course closing	- Final assessment and evaluation

**Figure 1:**
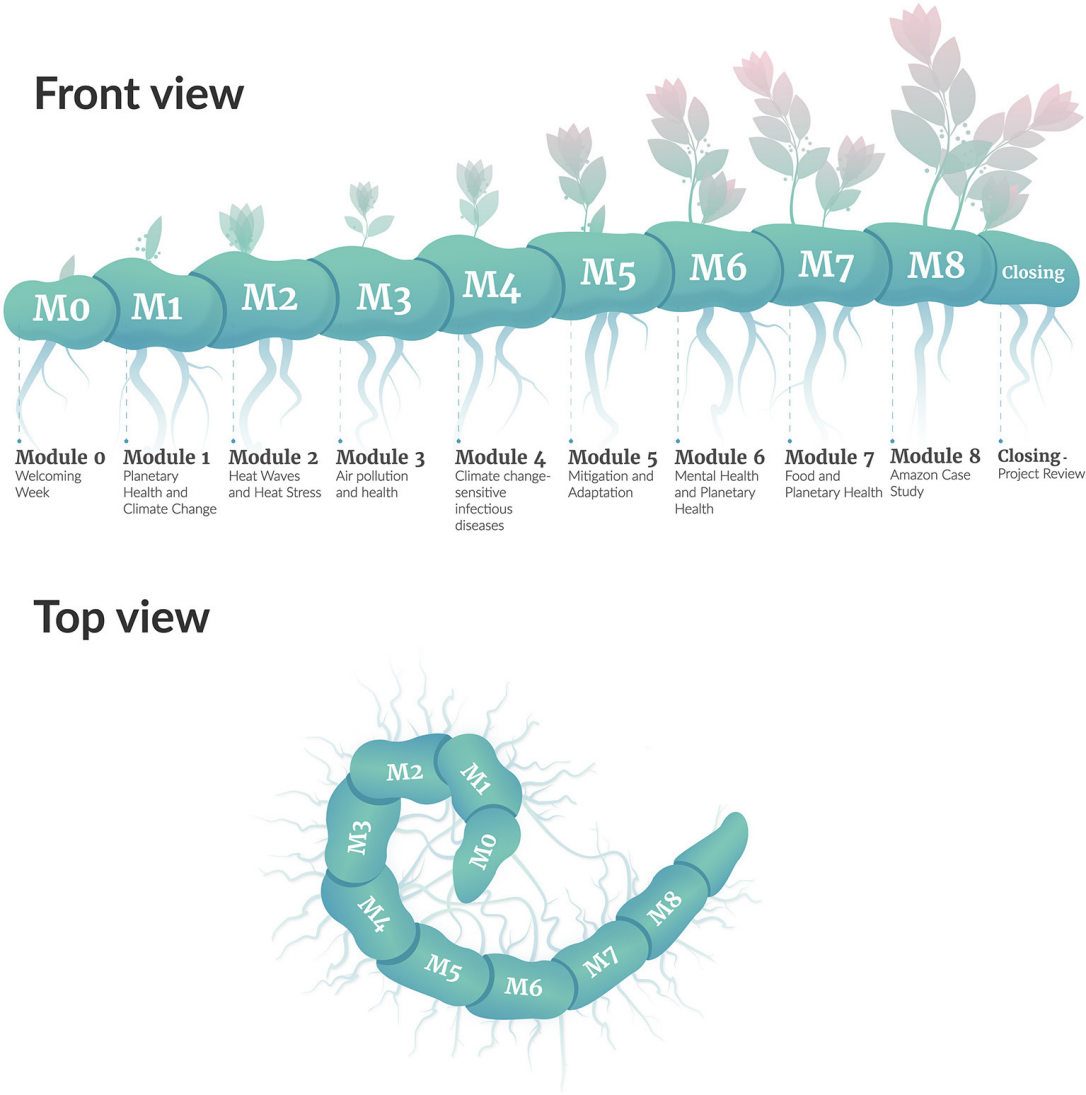
Rhizomes and rhizomatic course structure. Illustration credit: Iasmine Paim Nique da Silva and Lorenzo Costa Kupstaitis.

Selected articles, short videos, and podcasts deepened participants' understanding during the course. The course content and pedagogy was designed to be not only scientifically rigorous on the evidence, but also conscious of the intersections and *rhizomes*^1^ (Figure 1) of each theme. Less traditional activities and resources were included in an interactive forum, including debates, images, music, and documentaries.

**Figure 2:**
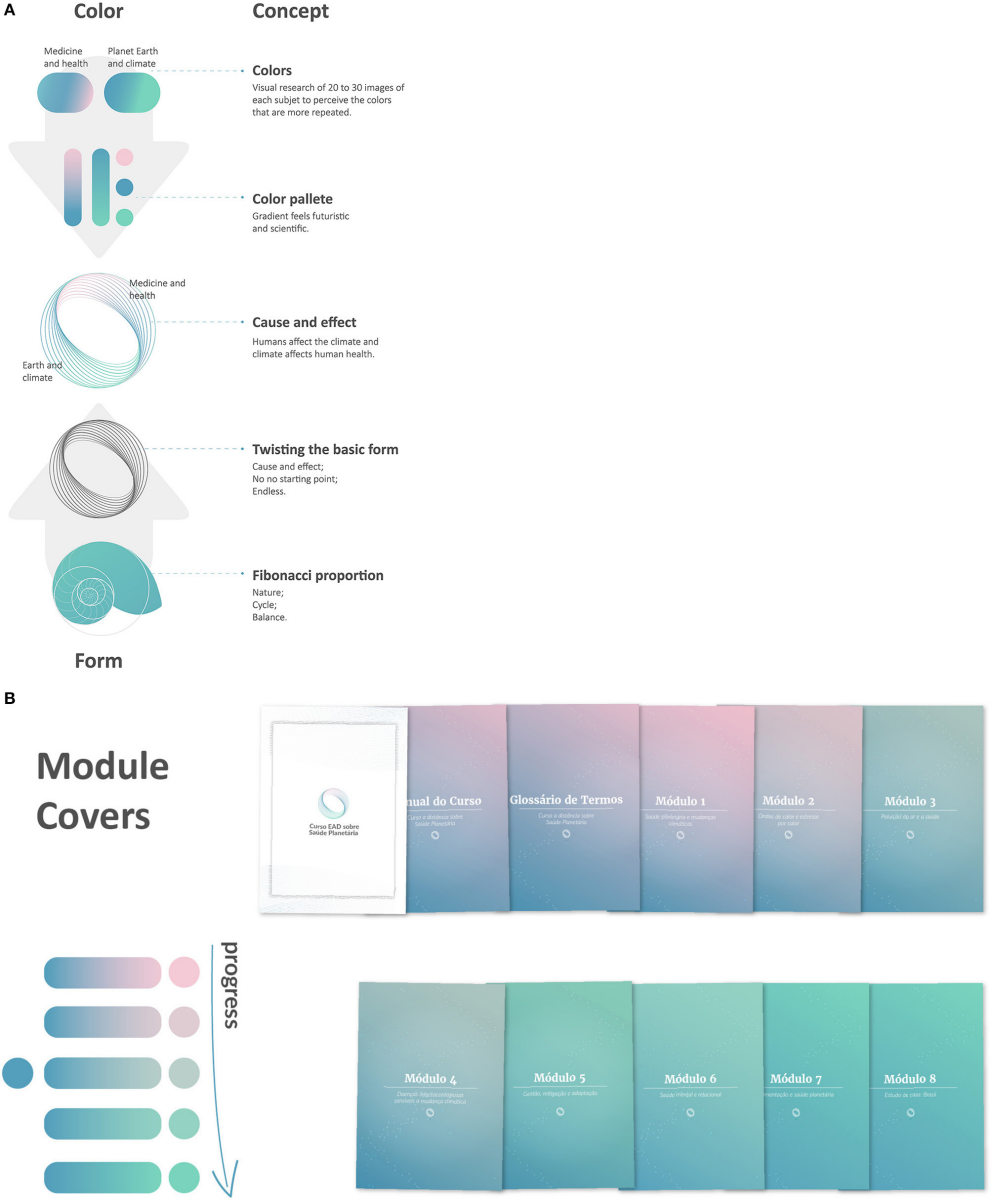
Art design structure and illustration of the Planetary Health MOOC with the Fibonacci sequence **(A)** and the palette colors choice **(B)**. Illustration credit: Iasmine Paim Nique da Silva and Lorenzo Costa Kupstaitis.

The course design was inspired by the image of organic life, taking shape naturally from strong roots. Students who completed all of the activities in each module received eight badges, with extra information. The badges were designed with references to Latin American Indigenous culture, to recognize their effort. Visual communication was key to an effective delivery of complex ideas. Course developers were continuously involved in the details of the course during the pilot, specifically focusing on how to simplify complex messages. The workload of the coordinators was especially dedicated to co-creation with the graphic designers. The TelessaudeRS-UFRGS design team collaborated with the course developers to represent planetary health visually through colors and form. The authors and designers researched images that represented health and the planet and consciously created a color palette for the course. The final image used the Fibonacci proportion in the course symbol to communicate the complex idea of cause and effect. These colors and ideas were applied to all course materials. The colors of the modules progressed throughout the course, leading the students from cause to effect. Forty images were illustrated, created, and designed for the entire course (Figure 2).

The MOOC course load had a total of 80 h, and, for the pilot, the modules were available weekly. The course was designed for low bandwidth internet and was able to run in the Modular Object-Oriented Dynamic Learning Environment (MOODLE), a free software to support learning in a virtual environment. Furthermore, four short animation videos were created in partnership with Rural Seeds, a group of young health professionals in training focused in rural areas. Additionally, three classes about central topics were recorded with TelessaudeRS-UFRGS. Supplementary Material was researched and selected from other sources like the Planetary Health Alliance. Many materials had to be translated from English as it was difficult to find Portuguese-language materials to use in developing the course. The selection of the topics was made through literature review, meetings with the course coordinators and thematic specialists. The course coordinators' own curiosity and professional experiences in Primary Care were also elements that contributed to the course content.

Initially, the target audience focused on health professionals. In November 2019, during the Third Porto Alegre Planetary Health Symposium, a young activist from Fridays for Future challenged the course creators to broaden the audience to anyone interested. At that point, the course was mostly structured, but the urgency to talk about planetary health at all levels presented a valuable opportunity. Following this, the group met and discussed how to make the course feasible for a larger audience, in order to at least reduce the knowledge gap in planetary health. The main target audience remained as professionals working in Primary Health Care. However, the course also opened to a wider, non-health audience. This was made possible by efforts at language simplification and by creating a glossary with 92 definitions related to Planetary Health.

**Figure 3:**
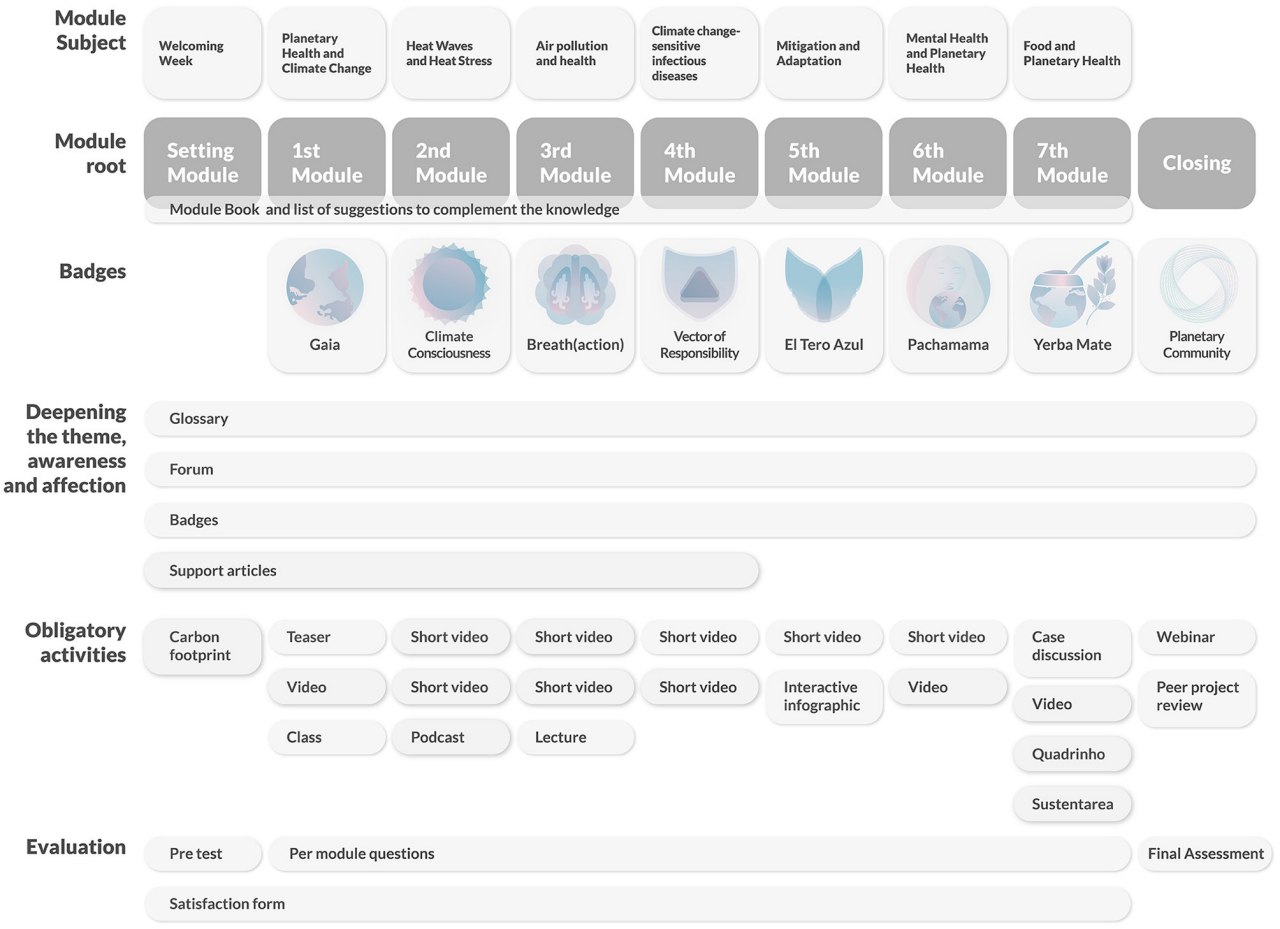
Content structure, root, and its deepening axis. Illustration credit: Iasmine Paim Nique da Silva and Lorenzo Costa Kupstaitis.

The content was organized in a way that gradually introduced the students to the topics. Every week, participants had an opportunity to explore each topic in depth. Terms, definitions, themes, and topics were presented in a gradual, stepwise fashion. The pilot was organized with a new module every week, with iterations and adaptations responding to the constant analysis of feedback received from students (Figure 3).

The course grading was done through module post-tests and a final assessment. These questionnaires consisted of five multiple-choice questions at the end of each module, totaling 60% of the students' final grade. The final evaluation was responsible for the remaining four points. For approval and certification, students needed to attain seven points out of ten. Before the course started, there was an ungraded pre-test to evaluate students' previous knowledge. User satisfaction surveys were used to evaluate the pilot after each module and the overall course. Analysis of the forum participation and submitted action plans provided qualitative information for evaluating the course. The course certification was granted by TelessaúdeRS-UFRGS, authenticated free of charge. We see this course as part of the fundamental right to education.

The course was launched on 4/27/2020 and concluded on 7/19/2020, during the COVID-19 pandemic and the deep economic, social, and environmental crisis that Brazil is experiencing. Even though the coordinators and creators were working as clinicians on the front lines, they still managed to follow the initial plan and timeframe for implementing the course.

By enrolling in the course, participants were invited to share data for the study and analysis of the pilot course, using an informed consent form. Data collection was approved by the ethics committee of the Grupo Hospitalar Conceição (CEP/GHC) (CAAE 25272619.7.0000.5530). This study analyzes the course database, profile of participants, answers to questionnaires, forum interaction, and submitted action plans.

## Results

### Profile of Participants

The course had a total of 2,777 participants enrolled. One thousand two hundred thirty-seven (44.54%) of them agreed to provide informed consent for this study (Table 2). As seen in Table 2, the majority of our audience was composed of women under the age of 35. Most of the participants were from Brazil, concentrated in the South and Southeast states. There were also four enrollees from Mozambique, one from Chile, and one from Peru. The professionals were mostly from areas related to health, mainly physicians, nurses, and dentists. However, other health categories were also represented. Interestingly, educators, computer scientists, and other non-health professionals also enrolled in the course. Regarding the workplace, we had notable participation of students and professionals from Primary Health Care. The youngest person that completed the course was 17 years old, and the oldest was 79 years old.

**Table 2:** Sociodemographic profile (*n* = 1,237).

**• Gender**	
	**Female: 879 (71.1%)**
	**Male: 352 (28.5%)**
	**Other: 6 (0.5%)**
**• Age**	
	** <18 years old: 22 (1.77%)**
	**19–25 years old: 347 (28.05%)**
	**26–35 years old: 416 (33.62%)**
	**36–45 years old: 248 (20.04%)**
	**46–59 years old: 162 (13.09%)**
	**>60 years old: 39 (3.15%)**
	**Not informed: 3 (0.24%)**
**• Profession/field**	
	**Medicine: 209 (16.90%)**
	**Nursing: 171 (13.82%)**
	**Nutrition: 115 (9.30%)**
	**Dentistry: 112 (9.05%)**
	**Biology: 76 (6.14%)**
	**Psychology: 53 (4.29%)**
	**Management/Administration: 47 (3.80%)**
	**Community Health worker: 41 (3.31%)**
	**Physiotherapy: 38 (3.07%)**
	**Pharmacy: 32 (2.59%)**
	**Nursing technician: 27 (2.18%)**
	**Physical education: 24 (1.94%)**
	**Social service: 23 (1.86%)**
	**Veterinary medicine: 23 (1.86%)**
	**Collective health: 17 (1.37%)**
	**Endemic combat agent: 16 (1.29%)**
	**Oral health technician: 12 (0.97%)**
	**Biomedicine: 13 (1.05%)**
	**Computer science: 10 (0.81%)**
	**Technical/General assistant: 10 (0.81%)**
	**Pedagogy: 6 (0.49%)**
	**Occupational therapy: 5 (0.40%)**
	**Other: 155 (12.53%)**
**• Place of work**	
	**Student: 389 (31.4%)**
	**Family health strategy: 278 (22.5%)**
	**Education: 159 (12.9%)**
	**Municipal health department: 114 (9.2%)**
	**Emergency care unit: 49 (4.0%)**
	**Others: 252 (20.0%)**
**• Education level**	
	**Graduation completed: 397 (32.1%)**
	**Post-graduate completed: 209 (16.9%)**
	**Undergraduate student: 158 (12.8%)**
	**Master's program completed: 93 (7.5%)**
	**High school: 79 (6.5%)**
	**Post-graduate in progress: 72 (5.8%)**
	**Medical or multi-professional residency in progress: 60 (4.9%)**
	**Master's program in progress: 53 (4.3%)**
	**PhD/Doctorate completed: 45 (3.6%)**
	**PhD/Doctorate in progress: 34 (2.7%)**
	**Medical or multi-professional residency completed: 34 (2.7%)**
	**Elementary/Middle school: 2 (0.2%)**

Around 24% of the participants were not from the health field (304), for example administration or biology. Of those enrolled, we had 155 (12.53%) of other professional areas, notably the field of environment (31), engineers (16), IT (10), and journalists (2).

### The Participants' Prior Knowledge and Awareness About Planetary Health

In the initial questionnaire, we asked what people understand by the term “planetary health,” we had 1,536 answers to this question. One hundred nineteen (7.70%) participants said that they did not know the theme; 105 (6.83%) explained it as “global health,” “world health,” or “one health.” The term “sustainability” appeared 142 (9.23%) times. Many people inferred the relation between human health and planet health. The terms “health of the planet,” “planet health,” or “Earth health” were mentioned 246 (14.83%) times without connection to human health. The word “man” appeared 43 times, on the other hand, the word “woman” was never cited—we expected that this might be an effect of “man” being taken to be synonymous with “humanity.” In the reviewed version, the terminology “man” was changed to “humanity” throughout the course material.

Further, we asked participants about where they gained their prior knowledge of the term “planetary health” (Table 3). Three hundred (37.3%) referred to knowledge from the internet, 109 (13.5%) from their formal education. Graduates were asked whether they already had practical or clinical content on planetary health. One thousand thirty-six (84.8%) participants did not have this experience. On the other hand, 99.1% thought of planetary health as an important or very important subject for students in the health professions to learn about it, and 1,132 (91.6%) believed that environmental changes, like climate change, would affect their patients' health. When asked about how they assess their workplace in terms of environmental sustainability, 395 (31.9%) responded that their communities and places of work are taking action. On the other hand, 449 (36.3%) responded that their workplaces carry out few actions. Of our participants, 365 (29.5%) felt alarmed, 784 (63.4%) concerned, 64 (5.2%) cautious, 13 (1.1%) indifferent, and 11 (0.9%) unconcerned regarding climate change. Seven hundred six (57.1%) of them answered that they describe themselves as connected to the environment, while 499 (40.3%) responded that they are both interconnected and committed to protection, 29 (2.3%) were indifferent, and 3 (0.2%) were disinterested.

**Table 3:** Previous knowledge and awareness about planetary health (*n* = 1,237).

	**This is necessary**	**This is useful, but not a priority**	**This is not necessary**	**I need more information before I decide**
How important do you think it is for health professionals to be able to understand the relationship between environment and human health?	1,096 (88.6%)	44 (3.6%)	5 (0.4%)	92 (7.4%)
How important do you think it is for healthcare professionals to be able to provide sustainable health care?	1,090 (88.1%)	51 (4.1%)	13 (1.1%)	83 (6.7%)
How important do you think it is for health professionals to be able to identify health problems associated with environmental problems?	1,136 (91.8%)	36 (2.9%)	4 (0.3%)	61 (4.9%)
How important do you think it is for health professionals to be able to adopt more sustainable practices in health services?	1,105 (89.3%)	58 (4.7%)	4 (0.3%)	70 (5.7%)
How important do you think it is for health professionals to be able to discuss what the duties of health professionals are toward planetary health?	1,093 (88.4%)	71 (5.7%)	4 (0.3%)	69 (5.6%)
How important do you think it is for healthcare professionals to be able to guide patients based on the effects of the environment on their health?	1,114 (90.1%)	57 (4.6%)	7 (0.6%)	59 (4.8%)

According to our questionnaire, 640 (51.7%) participants had never heard about Greenhouse Gas emitted by the health sector. When asked “how could you reduce it in your workplace?” referring to Greenhouse Gas emissions, 277 (22.4%) answered that they don't know what to do. Others replied with options such as using less paper, saving energy, using fewer anesthetic gases, properly separating garbage, or using fewer cars. 1,059 (85.6%) expressed their belief that the environment is changing and that there are ways to help, 167 (13.5) replied that they believe changes are occurring, but they don't identify initiatives that can help.

### The Forum

The forum was composed of 10 topics related to the modules. It was designed as a space for interaction between course participants to creatively share their reflections based on images, videos, short stories, poems, and songs. It was also a place for the participants to recommend readings and movies, share personal experiences, and disseminate projects they were involved with Table 4. Participants created and shared poems, music, and crafts. There were 237 interactions spread among the 10 topics, in which people committed to bring planetary health principles to their personal and professional lives, to mobilize local authorities, to be multipliers of the acquired knowledge, and even to introduce planetary health as a discipline in undergraduate medical courses. An example of a Forum week topic is presented in Box 1.

**Table 4:** Forum topics to encourage discussion, number of interactions, and key content/interactions.

**Topic**	**Number of interactions**	**Key content/interactions**
0. A contract with the planet	86	- Act as multipliers of acquired knowledge - Develop a pedagogical proposal for planetary citizenship - Introducing a discipline in an undergraduate medical program - Reduce, reuse, and recycle - Develop community gardens - Consume from local agro-ecological producers - Reduce energy and car consumption - Mobilize local authorities to carry out projects aimed at regeneration and sustainability - Commit to a change of conscience, with a unique perspective
1. How do your feet move in the world? What connections do you make between life and human health, climate change, and planetary health? Reflections about the WHO image campaign about climate change showing a foot connecting with the drought	46	- Life, human health, and planetary health are inextricably linked - Some people are more affected than others in a climatic emergency (those that have less impact) - We are all made of the same material—life—that returns in cycles - If we make the land suffer, those who live on it will also suffer
2. Short story and documentary about heat stress. Have you ever experienced a heat wave?	28	- Experience reports of how difficult it is to maintain activities during a heat wave, and when it is even worse for workers who already face poor working conditions - Reports of climate refugees who had to leave their lands due to the heat wave in the Brazilian State of Pernambuco
3. Is air quality measured in your region?	20	- Air quality measurements were found in some metropolises and all were above safe levels - Discussion on the use of masks to prevent particulate inhalation
4. COVID and planetary health: causes, consequences, and repercussions	21	- Deforestation affecting biodiversity and loss of ecological niches in wild animals—spillover (overflow) - Commercialization of wild species - Pollution as a factor that weakens and facilitates the development of the disease - Exposure of disadvantaged communities, without access to sanitation and conditions of social distance
5. What if you were a decision-maker? How do you suggest society and governments should face the climate emergency?	10	- Stimulate in the Brazilian Health System the principle of social participation and accountability of population - Admit the urgency of the problem
6. Earth Music and Environmental Racism. What feelings spring up in you?	10	- Hope vs. hopelessness - Minorities are the most affected - Inspire the discouraged and disbelievers to unite in favor of a new way of inhabiting the planet - Support those who are already in the struggle
7. Obstacles to sustainable food and how to overcome them	10	- Make your own food - Prepare booklets with guidelines
8. Art and planetary health	6	- A music playlist with songs that warn about the results of not caring for the environment - Handicraft ideas using seeds, leaves, and recycled material - A poem composed by one of the students
9. A contract with the planet. Where do we get?	0	–

Box 1 **Examples of Forum week topic for discussion.**

- Module 2—Heat waves and heat stress: Reflection on the short story and climate change.

***The Impossible World II*** (11)

*Brasília, September 26, 2084*.

“*Son, don't forget to put on your second skin,” says his mother*.

“*I already have it on, Mom” he answers*.

“*Are you already using your ultraviolet lens?” she asks from the kitchen*.

“*Yes, Mom, I don't want to burn my retinas,” he answers*.

“*And ...” says the mother, but the boy interrupts, “I already have my special clothes against ultraviolet rays and acclimatizing.”*

“*And …” his mother begins, “and the gloves too,” he finishes*.

*She follows, “Great, don't forget to bring water, don't get dehydrated.”*

*He responds, impatiently, “All right mom! I'm just playing soccer in the covered field, and then I'll be back.” As he speaks, he pulls on the oxygen mask and opens the door of the house*.

“*Okay, I just don't want you coming home with burns again,” says his mother, looking out the window*.

- Module 6—Mental and relational health: “In 1969, the composer Caetano Veloso was in prison in Rio de Janeiro when he received a magazine from his wife that contained the first photographs of the planet taken from outside the atmosphere. Admiring the globe from his cell, he was overwhelmed with emotion, which inspired him to compose the song ‘Earth,' in which he confesses to being in love with it. Listen to the song here (...).”

### Evaluation and Learning

Of the 1,237 participants who agreed to participate in the research, 614 (49.8%) completed the course, and of that subset 569 (92.67%) were accredited. Many participants completed the modules due to the fact that the access to the course pilot had a deadline and would be restricted afterwards in order to perform data analysis. The average final grade of students who completed the course was 8.9 out of 10.

The peer-reviewed evaluation consisted of developing an action plan that each student could apply in their own setting. The student could receive one extra point based on the project's evaluation, and another point for evaluating a colleague's project. A guide was provided to help students with writing their action plans. Students had 8 days to submit the one-page plan. Then, a randomized system allocated the submissions for peer review, which was open for the following 10 days. Two hundred forty-one plans were submitted, and the participants evaluated their colleagues' projects according to the following criteria: relevance of the proposal, stated guidelines, feasibility for putting into practice, and relation to planetary health. Peer-evaluators checked a box if the criteria were met and could add comments as needed. Key aspects of the action plans were highlighted in Box 2. Although the majority of the action plans were related to the Brazilian context, one of them was developed for Mozambique, related to hospital and primary care waste management at a national level.

Box 2 **Action plans perspectives and highlights (241 submissions).**

- The action plans concentrated on individual and community actions
- The community actions were more related to the area of Primary Care or schools
- Three major fields identified in the action plans were food and nutrition, infectious diseases, and garbage and recycling
- The majority of the action plans proposed the creation of vegetable gardens (in universities, health care units, schools, and communities); actions to implement garbage management and recycling, and interventions to prevent infectious diseases—mainly mosquito-borne diseases
- There were interesting proposals of reforestation and forest fire management and working with local rural communities to cultivate seeds (creating seed banks)
- Social media interventions and lectures
- Citizens and stakeholders committed to the plans for caring for the environment and individual changes. Undergraduate students were really active in the plans
- Deep reflections included changes in individual behavior and personal commitment like reducing meat consumption, reducing garbage and recycling, and consumption

At the end of the course, participants were invited to submit a video talking about their experience with the course, and 44 participants voluntarily took part. The videos had messages of gratitude about how much people learned from the course, and how key it was for changing their perspectives about how they see health and the environment. Selected key messages are available to watch with participant authorization.

### Satisfaction of Participants

The feedback questionnaire at the end of the course showed the satisfaction levels, with 65.5% feeling very satisfied; 31.8% satisfied; 0.4% dissatisfied; 1.9% very dissatisfied. Regarding the quality of the content, 74.4% responded that it was excellent; 22.2% good, and 3.2% considered the quality to be regular. When asked about the applicability of the topics explored regarding the participants' area of practice, 77% answered yes and 21.1% partially. 1.1% said they didn't apply. According to the overall evaluation of the course, using a nominal scale of 0–10, 46.9% scored the course as a ten; 34.6% scored the course as a nine; 13.2% as eight and 4.2% as seven.

Thus, of the people who answered this evaluation questionnaire, most of them felt at least satisfied, evaluated the course positively, approved of the content, appreciated the quality of the course, and saw the possibility of putting at least some of the content into practice. Notably, respondents concluded that the course changed their perspective on health, and sensed that their new openness to connections between health and the environment was irreversible.

### Feedback and New Versions

All the feedback and comments in the open questions were read by the team and analyzed to develop the next version of the course. A group of three people read all comments and feedback and organized comments by module on an electronic spreadsheet. Weaknesses were discussed and evaluated, for example quiz questions that seemed inaccurate were revised and sessions that were not working well were reviewed for improvement. For the English version, we also adapted content that was overly focused on Brazilian laws, so that it would be more appropriate and useful for an international community, also reflecting the feedback coming from pilot course participants from other countries.

The learners suggested many new modules for the course: water; the Amazon and cosmovisions; garbage and recycling; impacts of plastics in human health; xenobiotics/pesticides and their impacts on human health. It was also suggested that perspectives from schools and younger students should be included. There were also requests for aspects of urban design and architecture to appear more in the content of the modules.

The learning process from the pilot was consolidated with a team that read, analyzed, and discussed all the rich feedback we received from pilot participants, with possible changes and adaptations in mind. This informed the revised structure published in 2021, including revisions to quiz questions. One interesting feedback concerned the use of the word “homem” (men in English) referring to humanity (for example “man made”), as a gender issue. Generally, participants also want more examples of places that changed and actions. Participants also suggested two new topics for modules: water and indigenous knowledge. The team have subsequently created a new optional module for the 2021 about water and an optional module about indigenous knowledge and planetary health is also being planned. Another identified barrier was the tendency for videos to be in English, though Portuguese subtitles were provided.

## Discussion

The older generation is failing to provide the current and future generations with a healthy environment. The idea of globalization and technological achievements were responsible, in many ways, for humanity being understood as detached from nature and not as part of it. To change culture and “go down to Earth” (12), as the philosopher Bruno Latour wrote about reorienting politics in the Anthropocene, people need education. In this sense, learning about Planetary Health is pivotal for change.

An affective pedagogical framework is needed, to underpin a relational psychosocial approach which emphasizes the role of affective and unconscious processes in shaping engagement with the climate crisis (13). Current research on global citizenship and education for sustainable development (ESD) indicate that emotional, creative, and affective dimensions are key to transformative education (10). The context for teaching and learning has changed profoundly since 2019 when 1.6 million mostly young people mobilized as part of the “Fridays for Future” global action. The affective aspect of the course sought to recreate that level of engagement, particularly in the living process of building an engaged, politically active, ethical, and therefore, critical education (13). The planetary Health MOOC effort was shaped to at least partially address the disjunction between individuals' awareness of the climate crisis and daily behaviors that increase the climate crisis, which is a great paradox in education about climate change, and certainly planetary health (14).

A question that the authors had, employing the Freirean perspective, was: can we love the world again? (13) We do not have the answer to this question, but we wanted to engage people with science, affectivity, and experience, which is certainly a challenge in distance learning education. The MOOC in Planetary Health was created in Portuguese and helps to address the lack of training materials for other Lusophone countries, as it was a challenge to find and organize appropriate resources in Portuguese.

Our sample of participants was composed of 71.1% women—this predominantly female presence may deserve further qualitative investigation, and reflection, as gendered norms seem to be connected to nurturing roles involved in caring for the planet. Of the total number of participants, 62.81% were under 35 years old, showing how this theme is especially attractive for youth and could be further explored and developed for this demographic.

Studies of Northern and elite MOOCs show more uptake and benefits for already-privileged groups, particularly men and those already holding higher qualifications. This shows how MOOCs enhance individual career benefits, rather than collective public transformation for justice (15, 16). However, a distinctively social justice perspective on Open Education exists as a driver of non-elite MOOCs. These re-orient MOOCs toward the concept of open educational practices specifically to factor in social justice and inclusion (17). Arguably, Global South educators, information and communications technologists, and health and environmental activists have been continuously collaborating to bring together education, environment, and health activism with a social justice lens since the very beginning of the digital era in the 1980s (18). MOOC design is thus not merely technical but enacts different orientations and ways of being (19) and reflects the recently published framework to guide planetary health education (20). For example, digital neocolonialism (21) might be reinforced by MOOCs rather than MOOCs being a tool to challenge and transform unequal and unhealthy relations. Reversing this insight, can a MOOC designed to bring about transformative learning embody this in its design? Studies of elite Northern MOOCs point to problems with student engagement and low completion (22), leading to a drive for technological interventions to improve engagement (23). This Southern MOOC showed much higher engagement, completion rates and learner satisfaction.

There is hope that MOOCs offer a more “sustainable” form of education, however educational interventions' own environmental impacts are rarely researched (24). Conventional students already use digital technologies, infrastructure, and energy on a daily basis. Where MOOCs increase access to education and lower costs without compromising educational quality, they promise greater social inclusion with lower economic and environmental costs per user. One study conducted by the UK Open University finds that online and ICT-enhanced distance teaching models had significantly lowered environmental impacts compared to face-to-face teaching by eliminating the need for student travel, residential accommodation, and facilities (25). Any evaluation of “sustainability” must balance the economic cost, social benefits, and environmental impacts. This MOOC has involved the considerable mobilization of social and natural resources, since it relies on social and educational effort, technologies, digital infrastructures, and energy use. The production and consumption of any mass educational intervention necessarily contributes to resource use, thereby potentially perpetuating patterns of social and environmental injustice through environmental depletion. There is no way to participate in, let alone change any existing health system without using resources and creating impacts that may harm planetary health. This is why module five includes a focus on identifying risks in the health system and services itself and finding ways to mitigate and adapt to them in the perspective of individuals, communities and policies.

The majority of the participants were concerned with climate change, trained in the health area, and worked in primary health care in places that did not often carry out sustainability programs. Significantly, there was a variety of professionals enrolled in the course with a range of educational levels, supporting the need for an open course offering. At the same time, the quite expressive undergraduate (12.8%) participation showed the demand for the topics discussed in this course to be brought into the college and university curriculum (26). Undergraduate, masters, PhD students, and residency trainees comprised 22.5% of the participants. The active search and the high enrollment of university students is illustrative of the gap that needs to be filled by Planetary Health discussions in the universities and formal health training programs. The vast majority of participants had completed or were enrolled in higher education (93.3%), though it is important to take note of the elementary and high school participants (6.7%), who might also represent a promising public.

The use of the planetary lens (27) and open perspective of the course centered the need to communicate planetary health topics to communities. The local plans reflected the motto of “think global and act local” and how to start reflecting about planetary health at an individual and local level, with participants proposing paths for change in areas like food, infectious diseases, waste management and consumption. The number of plans submitted (241) are also a marker of the success of the MOOC's pedagogy and engagement.

The forum participation was fundamental to deepen the roots of planetary health and spread its application to daily life. The need to broaden knowledge and recognize the importance of other rationalities, besides biomedical, were emphasized by the badges reflecting Indigenous Brazilian cultural expressions and stories.

Different MOOCs highlight different levels of critical thinking and practical demonstration. As Adam (28) shows, some course designs focus more on structural and systemic problems at a societal level and others at the community or individual level, reflecting different conceptualizations of (in)justice. Given our current context, we strongly agree that individual and community level behaviors may have limited transformative potential for the broader societal level, unless structural and upstream determinants are addressed, especially the behavior of powerful actors. However, these are immensely challenging to address and can be overwhelming for learners at the current moment and at our level of practice. Practical tools and small scale actions are important for connecting daily actions to the greater change that is needed and for maintaining survival and hope. This is not meant to displace a larger need for transformative critical consciousness and change by the powerful agents most responsible for compromising health at the planetary level.

The Brazilian social, political, and environmental context is one of massive deforestation, extensive fires and biomass burning, and changing land use critically affecting many biomes (Amazon, Pantanal, Cerrado, and others) (29). The human and ecological suffering that has resulted reflects forms of necropolitics (30) that are unfolding in the context of an infodemic and a coronavirus pandemic (31). These factors made this course a resource to target social determinants for health professionals and communities. The call to understand the urgency of the theme and the necessity for survival activism is crucial (32), even for the leaders/coordinators of this MOOC in Planetary Health. We nevertheless feel somewhat hopeful, even with Brazil suffering one of the biggest and most complex intersecting crises in its history, we were able to create the content and execute this course, reflecting our culture and stories, identities, and values to drive social and planetary health changes. “Survival activism” is a term that we have coined ourselves to describe an activist attitude that privileges people's well-being and survival despite the odds, and continuing to work within real limitations without entirely losing hope. This activism is one face of resistance to “necropolitics,” a term coined by the philosopher Achille Mbembe to describe situations where the life experience for a large mass of people is experienced as being so cruel that it might be called a “living death” (30). The story of this MOOC is also an example of the importance of civil society (universities, health professionals, and social movements) in occupying the challenging space and expressing the immediate need to respond to so many aggressions to the planet and its people. More than a reaction, this is a necessary proactive movement to create awareness and commitment, among health professionals and beyond, to put Planetary Health and the core of intersectoral health policies.

Moreover, this Planetary Health MOOC challenges North-South asymmetries and Eurocentric and Anglophone homogeneity of global open education resources by creating an alternative with a decolonizing perspective (33). Planetary health should be carefully, critically and reflexively approached, so it does not itself become a colonizing concept. In this sense, a “decolonizing perspective” is necessary to acknowledge the historical, economic, cultural, and ideological impact of colonialism and to seek to deconstruct unjust colonial ideologies and social arrangements including racism and Eurocentrism. A decolonizing perspective addresses both external and internal conditions of neocolonialism with the aim of ending and seeking reparation for colonial dispossession, violence, and inequity. This certainly shows the protagonism of Latin America and its generative response to the call for quality education for all. We reiterate that education is a human right and we maintain the belief that this MOOC should be free of charge, with free certification for all citizens of the world (34) who successfully complete the course.

The positive course feedback was decisive for understanding the importance of rooting knowledge within an evidence-based approach, but also with art and a diversity of learning tools and processes (podcasts, reading, video, etc.). In the context of the coronavirus pandemic, the course availability was certainly a timely effort to expand free online education. The MOOC in Planetary Health is a good pivot for thinking about expanding access to relevant information. Finally, we highlight the main characteristics of the course—multidisciplinary, innovative, provision of accessible knowledge, theory, advocacy, traditional and indigenous knowledge recognition, and implication of its participants—as key for framing this experience as a successful and inspiring one.

## Future Steps

The course was relaunched in 2021 with the incorporation and analysis of more than one thousand suggestions. From December 2020 until December 2021 the course will be available in the TelessaúdeRS-UFRGS platform in the Brazilian Portuguese version, and an English version was made in partnership with Planetary Health Alliance. A new version of the course was created by WONCA Environment focused on Primary Care and it was launched in 2021. The authors envision training as many health workers and members of the public as possible, recognizing their right to education and their readiness to learn and increase their awareness of planetary health topics.

In terms of research, we recognize the importance of more in-depth evaluations, especially qualitative data, to better understand the potential of specific publics (e.g., young women, different geographical audiences) and explore the global-local connections, especially through the action plans.

## Limitations

The unequal access to the internet and digital information is probably one of the biggest limitations of this course. MOOCs in the Global South face additional challenges of the digital divide on top of recognized problems of low retention and completion. Although this MOOC showed fairly strong participation and completion, considering global comparisons, we need to keep in mind that MOOCs tend to reach more privileged people with better connectivity and resources (21). Participants need a range of literacies to benefit from MOOCs, even basic computer literacy and interaction between digital spaces is an essential component, from an educational perspective (21).

The scope of this paper is limited to the survey findings of the pilot MOOC. Advocacy, policy and movement building are essential concerns for mobilizing PH generally, and this is reflected implicitly in the collaborative partnerships that led to the design and rollout of the pilot MOOC. However, this broader discussion would be best addressed in a separate article addressing that scope.

The course team became aware that incorporating indigenous perspectives is a collaborative learning process that requires much more space and time to experience, taking care to avoid a superficial treatment of the subject. A new module will be created involving a number of indigenous leaders in its design. All new modules start as optional (e.g., the water module) and can only become obligatory in subsequent iterations of the course.

The language of the MOOC remains concentrated on health, which may deter people from other disciplines. Nonetheless, we had participants from different professions and at various educational levels. The pilot launched during the coronavirus pandemic, which was previously considered as a possible limitation, but it may have been an enhancement and possibly increased participation. The short timeframe for concluding the MOOC affected the number of people that completed it.

Only 50% of consenting participants completed the MOOC, including the survey that this article is based on (619/1,237). This could be seen as a limitation, however inclusion of the survey as a condition for completion maximized the opportunities to learn from the pilot and consider improvements for future adaptations of the course.

## Data Availability Statement

The raw data supporting the conclusions of this article will be made available by the authors, without undue reservation.

## Ethics Statement

The studies involving human participants were reviewed and approved by data collection and analysis was approved by the Ethics Committee of the Grupo Hospitalar Conceição (CEP/GHC) (CAAE 25272619.7.0000.5530). This study analyzes the course database, profile of participants, answers to questionnaires, forum interaction, and action plans submitted. The patients/participants provided their written informed consent to participate in this study.

## Author Contributions

MF, CV, YR, AC, AB, and MG designed data collection tools, monitored data collection for the whole study, wrote the analysis plan, cleaned and analyzed the data, and drafted and revised the paper. MF and CV were the coordinators. DA, EF, CF, ED, PS, S-MK, and MG analyzed the data, and drafted and revised the paper. All members of the Planetary Health MOOC group (available in the Table 1 in the Supplementary Material) designed the course and supported the data analysis of this paper, the index included only the authors that agreed to take part in this study. All authors were responsible for ensuring the accuracy and integrity of their contributions and approved the final manuscript.

## Funding

The analysis and course creation/coordination was volunteer-based. TelessaúdeRS-UFRGS worked with its educational platform and team without any specific funding for the project. We gratefully acknowledge the Institute of Advanced Studies, Universidade de São Paulo, São Paulo for financial support to cover the article processing charge for this article, and Frontiers in Public Health for applying a fee reduction.

## Conflict of Interest

The authors declare that the research was conducted in the absence of any commercial or financial relationships that could be construed as a potential conflict of interest.

## Publisher's Note

All claims expressed in this article are solely those of the authors and do not necessarily represent those of their affiliated organizations, or those of the publisher, the editors and the reviewers. Any product that may be evaluated in this article, or claim that may be made by its manufacturer, is not guaranteed or endorsed by the publisher.

## Footnotes

^1^A horizontal, usually underground stem that often sends out roots and shoots from its nodes.
