# Supplementary material for: Development and Assessment of a Brazilian Pilot Massive Open Online Course in Planetary Health Education: An Innovative Model for Primary Care Professionals and Community Training
Source: Front Public Health. 2021 Dec 15;9:663783. doi: 10.3389/fpubh.2021.663783 (PMC8715956; doi:10.3389/fpubh.2021.663783)
Supplement: Supplementary file 1 [file Data_Sheet_1.docx]

**Supplementary Material**

**Consortium/group**

**Development and assessment of a Brazilian pilot Massive Open On-line Course in Planetary health Education: an innovative model for primary care professionals and community training**

**Authors**

Mayara Floss; Carlos Augusto Vieira Ilgenfritz, Ylana Elias Rodrigues; Anna Cláudia Dilda; Ana Paula Borngräber Corrêa; Diego Azevedo Conte de Melo; Enrique Falceto Barros; Carlos Alberto Faerron Guzmán; Erin Devlin; Paulo Hilário Nascimento Saldiva; Su-Ming Khoo; Marcelo Rodrigues Gonçalves and the Planetary Health MOOC group

**Supplementary Material**

In May 2019, we started the project to build the Massive Open Online Course pilot on planetary health with the presentation of the proposal to the Telessaúde team in August 2019. The development of the instructional design and methodology of the course began to be elaborated, as well as approval by the ethics and research committee of Grupo Hospitalar Conceição. As soon as it was approved in October 2019, the coordination of the course began to invite renowned authors in planetary health in Brazil and Latin America in several areas of research and knowledge to elaborate the content of the modules and start their development.

The Planetary Health MOOC would not be possible without the help of its all contributors, specially the TelessaúdeRS-UFRGS where it was developed. A total of 59 thought partners, designers, professors, researchers and health professionals involved in education efforts at the intersection of health and environmental change from around the world participated in the course creation, from content development to artistic and instructional design. They also helped in the data analysis, findings, and knowledge systematization of this course.

**Planetary Health MOOC group**

Airton Tetelbom Stein, Aline Martins de Carvalho, Andressa Cavalcante Paz e Silva, Andrews Vieira Berni, Angélica Dias Pinheiro, Amber Wheatley, Ana Célia da Silva Siqueira, Bianca Cadore Morás, Bianca Niemezewski Silveira, Bruna Marmett, Camila Alscher Kupac, Camila Giugliani, Camila Hofstetter Camini, Camila Vescovi, Carlos Dora, Carolina Zanette Dill, Carolline Paggi Montemezzo, Cláudia Ramos Rhoden, Constance Oderich, Davi Perin Adorna, Dirce Maria Lobo Marchioni, Eno Dias de Castro Filho, Fernando Xavier, Geise Ribeiro da Silva, Héctor Gonçalves Lacerda, Iasmine Paim Nique da Silva, Jacqueline Ponzo, Karina Pavão Patrício, Laíse Andressa de Abreu Jergensen, Laura dos Santos Boeira, Lorenzo Costa Kupstaitis, Luís Gustavo Ruwer da Silva, Marcela Araújo de Oliveira Santana, Marco Aurélio Ferreira, Marilyn Urrutia Pereira, Nelzair Araujo Vianna, Nicoli Bonalume, Olga Garcia Falceto, Paola Rava Dellepiane, Paulo Henrique Arantes, Rafaela Brugalli Zandavalli, Roberto de Almeida, Roberto Nunes Umpierre, Rodolfo Souza da Silva, Roseana Boek Carvalho, Sandra de Souza Hacon, Sérgio Sirena, Tatiana Souza de Camargo.
